# Habitat selection and influence on hunting success in female Australian fur seals

**DOI:** 10.1038/s41598-024-78643-5

**Published:** 2024-11-06

**Authors:** Saia Nahir Bartes, Jacquomo Monk, Chris Jenkins, Mark A. Hindell, Daniel P. Costa, John P. Y. Arnould

**Affiliations:** 1 School of Life and Environmental Sciences, Deakin University, 221 Burwood Highway, Burwood, VIC 3125 Australia; 2 Institute for Marine and Antarctic Studies, University of Tasmania, Hobart, TAS 7001 Australia; 3 Institute of Artic and Alpine Research, University of Colorado Boulder, Boulder, USA; 4 School of Biological Sciences, University of Adelaide, Adelaide, Australia; 5 Department of Ecology and Evolutionary Biology, University of California Santa Cruz, Santa Cruz, CA USA

**Keywords:** Habitat selection, Hunting success, Australian fur seals, Ecology, Animal migration, Behavioural ecology, Conservation biology, Population dynamics

## Abstract

Determining the factors influencing habitat selection and hunting success in top predators is crucial for understanding how these species may respond to environmental changes. For marine top predators, such factors have been documented in pelagic foragers, with habitat use and hunting success being linked to chlorophyll-*a* concentrations, sea surface temperature and light conditions. In contrast, little is known about the determinants of benthic marine predators. The Australian fur seal (*Arctocephalus pusillus doriferus*) is a benthic-diving forager that has a breeding and foraging distribution largely restricted to Bass Strait, the shallow (max. depth 80 m) continental shelf region between the Australian mainland and Tasmania. The species forages mostly on benthic prey and represents the greatest resident marine predator biomass in south-eastern Australia. The region is also one of the world’s fastest-warming marine areas and oceanographic changes are influencing shifts in prey distribution and abundance. In the present study, GPS-derived locations of benthic dives (*n* = 288,449) and dive behaviour metrics were used to determine seafloor habitat selection and factors influencing hunting success in 113 lactating adult females from Kanowna Island during the winters of 2006–2021. Individuals non-randomly selected foraging habitats comprised of deeper, steeper sloped, muddy-sandy areas with less gravel and highly disturbed regions (*P* < 0.01). Hunting success was greatest in shallower rocky reefs (< 30 m) and deep areas (> 40 m) characterised by moderate presence of gravel (25–50%) and substantial rock composition (50–75%) on the seabed. These findings suggest that habitat use and hunting success in adult female Australian fur seals could be impacted by predicted oceanographic changes, such as rising temperature, altered currents and waves which may modify seafloor characteristics and benthic communities.

## Introduction

Habitat selection is the active process through which organisms select a potentially appropriate area determined by a combination of genetic and behavioural factors, guided by specific environmental cues^1,2^. This process is critical for understanding the complex behavioural and environmental conditions that influence the survival and fitness of individuals and is a central focus in ecology^3,4^. Some common features influencing the selection of a suitable habitat include the sex and age of the individuals^5^, the availability and abundance of food^6^, prey catchability^7^, provision of shelter, protection against predators, and other characteristics that facilitate the locomotion of individuals^2^. There are other less-studied factors that can also impact habitat selection, and unravelling their influence is equally important for ecologists.

Given the low probability of all necessary resources for an organism being uniformly distributed in space, the limiting resource required for each activity will be the driving force in habitat selection^8,9^. Consequently, habitat selection will depend on habitat features necessary to carry out the specific activity at a given moment. For instance, the short-term decision to choose a foraging habitat depends on balancing an animal’s activity budget while managing time and energy constraints, which are further shaped by factors such as physiological capacity, phenology, predation risk, competition, and the spatiotemporal distribution of food resources^10^. All of these factors ultimately influence foraging success^11^. In contrast, the long-term selection of a new breeding site depends on the essential reproductive components required for successful breeding e.g. finding a suitable partner and low interspecific competition^12^.

The selection of a suitable foraging habitat involves choosing an optimal region to ensure a high likelihood of foraging success. Hunting success is fundamental in predators survival and has also been correlated with reproductive success^13^. The determinants influencing the selection of optimal foraging areas differ between trophic levels and environment types^14^. For top predators, foraging areas with high hunting success have been related directly or indirectly to prey abundance, availability and reduced interspecific and intraspecific competition^14^. Conversely, the key determinant for prey is to occupy areas that include habitat features that comprise predator search rate and/or capture efficiency^14^.

Studies on habitat selection and hunting success in terrestrial predators have been facilitated by the ability to directly observe prey consumption and its relationship to environmental parameters. These direct observations have revealed that factors such as air temperature, vegetation structure, light, moon phases, and wind speed influence habitat selection and optimise hunting success in predators^15^. For example, direct observations have shown that long grass and dense shrubs benefit the selection of foraging areas and improve the hunting success of lion *Panthera leo*^15^. In marine environments, studies have relied on indirect observations based on tracking technologies^16–18^, which have shown consistent links to some environmental factors that are proxies for prey availability, and these factors can differ between pelagic and benthic predators. For pelagic predators, key factors that influence habitat selection and optimise hunting success include sea-surface chlorophyll-*a* concentration, sea surface temperature, and light conditions^16–18^. In contrast, benthic predators are affected by different factors such as seafloor sediment composition, turbidity, and bathymetry^19–21^. These environmental factors are expected to undergo significant changes due to climate change, with stronger wind-induced currents and wave activity likely increasing seabed sediment erosion and mobility^22,23^ and rising temperature affecting prey distribution^24–27^. Such alterations can modify species distribution which effects can scale up to top predators, potentially impacting their selection of foraging areas and success^28^. Therefore, identifying the environmental predictors that best explain patterns of hunting success and marine habitat use is important for understanding the role of these species within the ecosystem and predicting their response to anticipated environmental changes.

The Australian fur seal (*Arctocephalus pusillus doriferus*; AUFS) is a primarily benthic-diving forager comprising the greatest resident marine predator biomass in south-eastern Australia^21,29–31^. The species is a generalist predator, consuming a large array of prey(> 70 taxa^32,33^), that are predominantly benthic, with some pelagic species, and exhibits seasonal dietary variation that correlates with prey availability and its reproductive cycle^32,34,35^. Foraging occurs primarily on the sea floor, where benthic/demersal species such as red cod (*Pseudophycis bachus*), leatherjackets spp. (Family Monacanthidae), barracouta (*Thyrsites atun*) and octopus (e.g. pale octopus *Octopus berrima/pallidus* and maori octopus *Octopus maoruma*) are targeted during the summer. In contrast, coastal pelagic species frequently occurred in the winter diet with items like flat imperator (*Beryx decadactylus*), jack mackerel (*Trachurus sp*) and ocean jacket (*Nelusetta ayraudi*^33,36,37^). Preliminary studies on habitat selection and hunting success on AUFS have shown correlations with sea surface temperature and chlorophyll-*a*^21,38^, factors expected to be modified by climate change.

The breeding and foraging distribution of AUFS is largely restricted to Bass Strait, the shallow (maximum depth 80 m) continental shelf region between the Australian mainland and Tasmania^39,40^, which is located near their main colonies. Females foraging behaviour is limited to the nutrient-poor waters and low marine primary productivity of the Bass Strait region^41,42^, due to the need to frequently return to provision their offspring^18,19^. This region is also one of the fastest-warming oceanic areas in the world^43,44^, with anticipated oceanographic changes, such as the strengthening of wind-induced currents and wave activity^22^, expected to modify the distribution, diversity and abundance of species^24–27^. These changes are likely to impact the foraging habitat of the AUFS^21^, as shifting currents may alter turbidity and seafloor sediment composition^22,23^, affecting the benthic environment they feed on. While the effect of some environmental variables on habitat selection and hunting success has been documented, there is currently limited information on how seafloor factors influence the selection of optimal benthic foraging grounds in AUFS, despite benthic dives being the predominant foraging behaviour in this species^21^. This knowledge gap makes it challenging to predict the impact of anticipated oceanographic changes on their primary benthic and demersal foraging habitat.

Previous studies have suggested that seafloor features are crucial in shaping foraging behaviour in top predators^19,45^ and are, therefore, expected to have a significant influence on AUFS. Given the potential impact of climate change on these factors, the present study aimed to determine the benthic environmental factors influencing: (i) habitat selection; and (ii) hunting success in adult female Australian fur seals provisioning pups. This information was subsequently used to predict optimal foraging areas and assess how future environmental conditions might influence hunting success in AUFS.

## Results

**Fig. 1:**
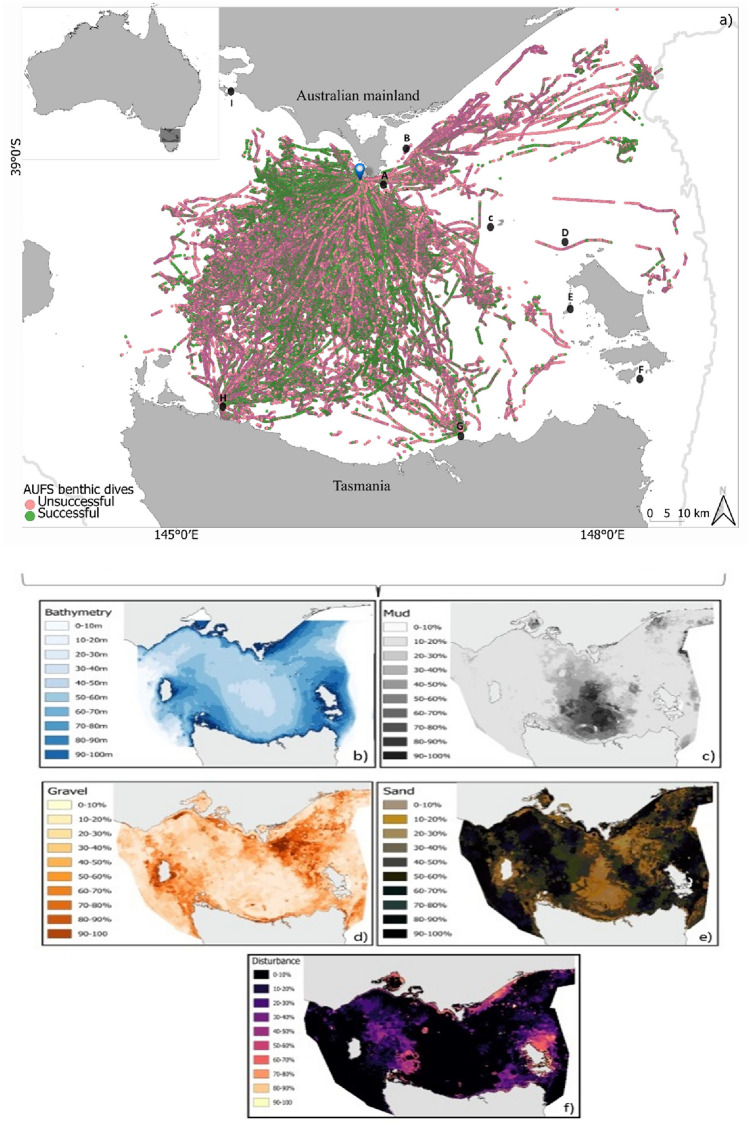
The foraging areas of adult female Australian fur seals from Kanowna Island (blue location marker) in Bass Strait, southeastern Australia, with the locations of successful (green) and unsuccessful (pink) benthic dives (**a**) and the most significant environmental variables in their foraging habitat selection (**b**-**f**). Australian fur seal colonies are indicated by black location markers: A: West Moncoeur; B: Rag Island; C: Judgments Rocks; D: Wright Rocks; E: Double Island; F: Moriarty Rocks; G: Tenth Rock; H: Bull Rock; I: Seal Rock. The grey line represents the continental shelf, isobath 200 m.

At-sea movements and diving behaviour data were collected from 113 adult females at the breeding colony of Kanowna Island. The number of deployments each year varied from 2 to 16 individuals between 2006 and 2021, resulting in a total of 288,449 dives recorded during multiple foraging trips per individual (Table 1; A2). Females predominantly travelled throughout the central Bass Strait, where approximately 90% of all dives occurred (Fig. 1). However, some individuals foraged near the Tasmania coast and several others travelled northeast of Kanowna Island (Fig. 1).

**Table 1:** Summary of the deployments of Australian fur seal females at Kanowna Island from 2006 to 2021.

Year	Seals (n)	Benthic dives (n)	Depth range (m)	Successful dives (%)
2006	2	4941	40.5–83.5	44.3
2007	2	3291	40.5–86	16.5
2008	9	8868	40.5–89	31.4
2009	14	24,414	4.5–124.5	38.0
2010	4	3138	20.5–83	32.1
2011	8	12,490	4.5–95	36.7
2012	14	11,961	5.5–86.5	43.0
2013	7	24,172	5.5–89.5	56.6
2014	4	14,029	20.5–87.5	50.4
2015	3	4269	30.5–83.5	34.4
2016	3	4285	40.5–87	17.93
2017	8	12,872	40.5–86.5	37.8
2018	6	12,206	20.5–86.5	25.5
2019	7	56,413	40.5–93.5	59.3
2020	16	61,641	4.5–89.5	47.88
2021	6	29,459	40.5–87.5	51.5

**Fig. 2:**
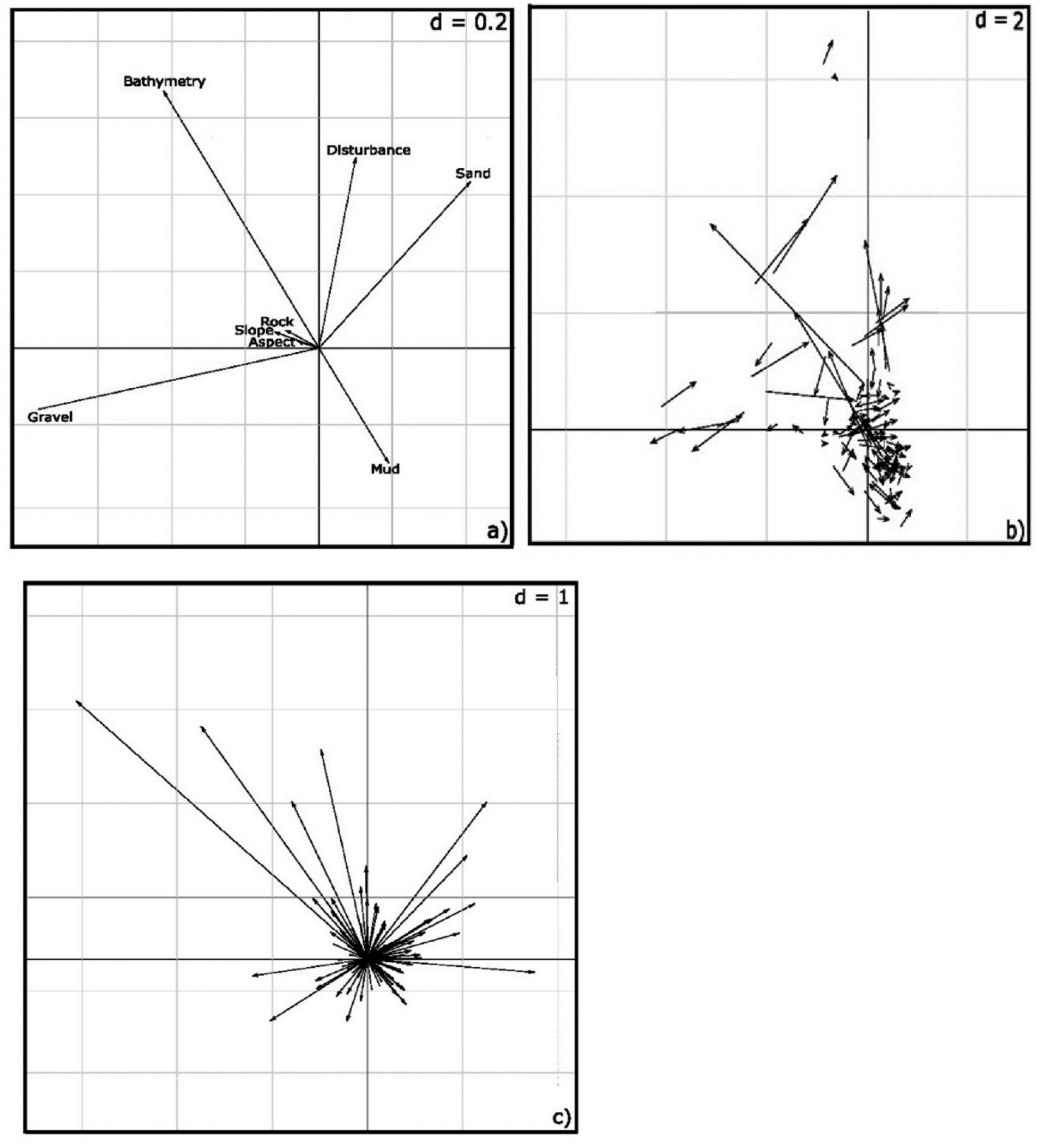
Habitat selection by 113 adult female Australian fur seals from Kanowna Island. (**a**) variables loadings on the two first factorial axes (axis 1: *x axis*; axis 2: *y axis*) , (**b**) the marginality vectors of individuals on the first factorial plane, the end of the arrows correspond to the mean characteristics of the habitat on the relocation of individuals, and (**c**) the marginality vectors of individuals after re-centering each home range composition which is axis 1 and axis 2.

The *K*-select analysis resulted in the first two axes accounting for 73% of the marginality of individuals. The randomization test conducted on the marginality vectors revealed habitat selection was significantly non-random for all the animals (*P* < 0.05, Fig. 2a). Individuals selected foraging habitats comprised of deeper, steeper sloped, muddy-sandy areas with less gravel and highly disturbed seafloor (*P* < 0.001; Fig. 2b). Some females exhibited similar preferences for habitat characteristics by selecting deeper areas or regions with greater mud content or choosing locations with increased bottom floor disturbance and sandy substrates. However, the females had no distinct preference between rocky or sandy seabed structure (Fig. 2c).

**Fig. 3:**
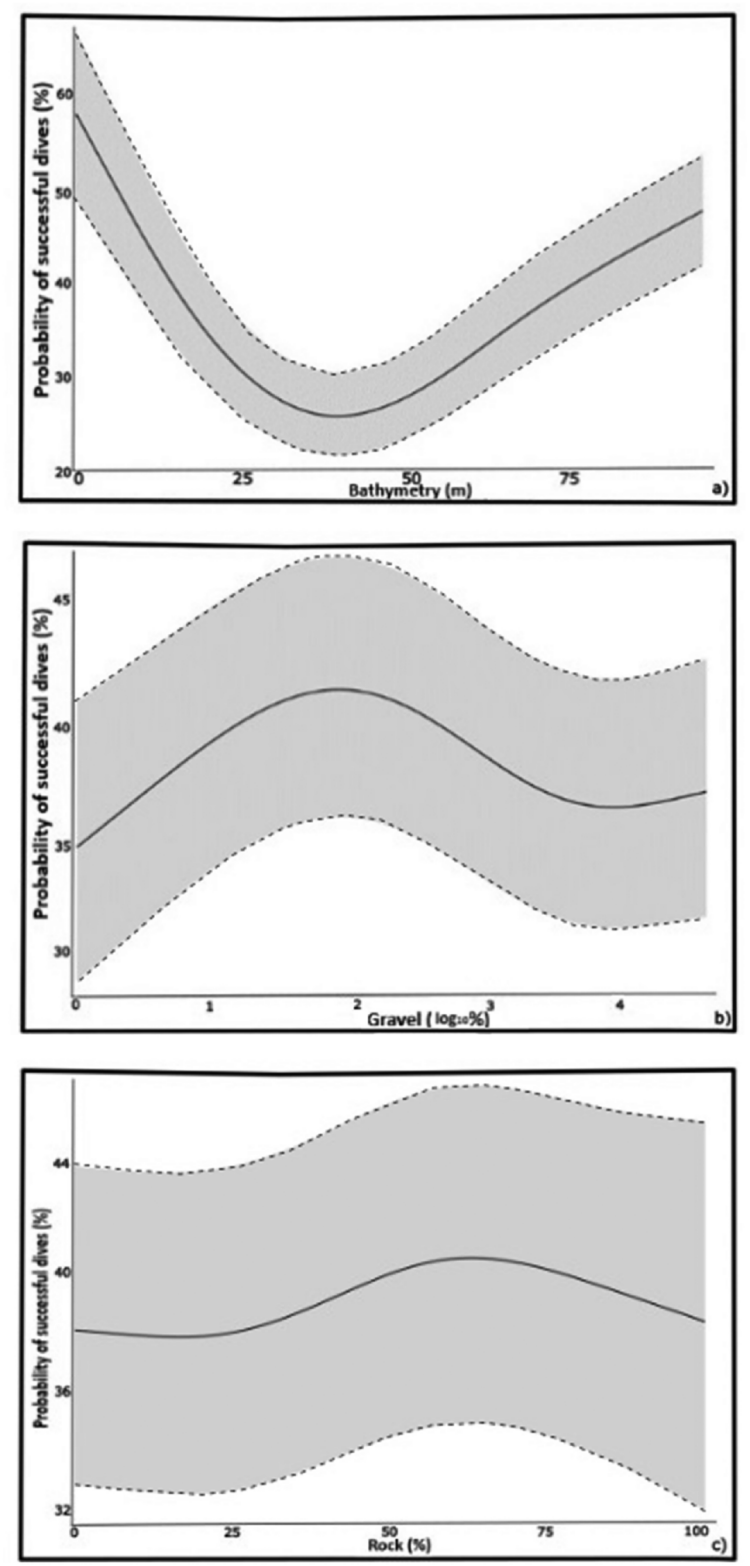
Relationship between the benthic environmental variables of bathymetry (**a**), gravel cover (log_10%_; **b**) and rock (**c**) and hunting success in 113 adult female Australian fur seals.

**Fig. 4:**
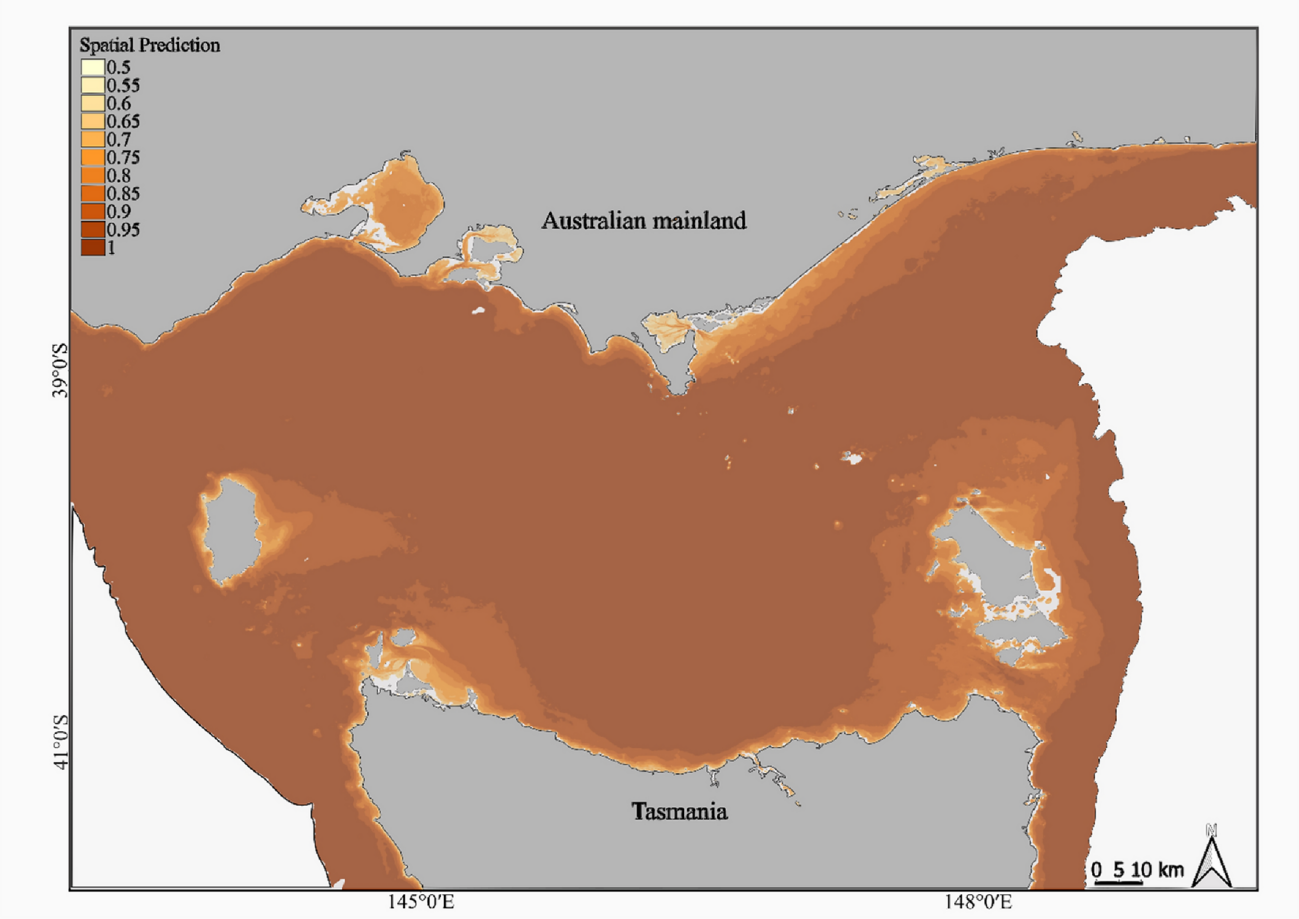
Spatial prediction from a general additive mixed effect model of successful foraging areas for adult female Australian fur seals from Kanowna Island. Darker shades of orange indicate areas of predicted greater hunting success based on the important variables derived from the model.

The dive classification indicated that 47% (*n* = 136,080) were successful. Out of the 63 models fitted in the FSSgam test, the most parsimonious model identified bathymetry, gravel, and rock as the most influential variables in determining hunting success. These factors collectively explained the highest variation in the model subset, suggesting AUFS exhibit greater success in benthic dives when encountering these habitat features. The GAMM outcomes revealed that hunting success in AUFS was greatest in shallower waters (< 30 m; Fig. 3a) and deep areas (> 40 m), characterised by the moderate presence of gravel (25–50%; Fig. 3b) and substantial rock composition (50–75%) on the seafloor (Fig. 3c; P < 0.001). The spatial prediction derived from the GAMM predictors indicated that the central and northeast regions of Bass Strait have the most conducive habitat conditions for hunting success for AUFS (Fig. 4).

## Discussion

Habitat selection by predators is a crucial process in shaping ecological communities and maintaining ecosystem function^46^. The selection of a foraging habitat influences hunting success and, ultimately, individual survival^47,48^. Understanding the factors affecting both parameters in top predators is essential for predicting their responses to environmental changes. The present study investigated how benthic habitat features impact habitat selection and hunting success in adult female AUFS provisioning pups. The results reveal that female AUFS exhibited a non-random selection of foraging areas, with preferences for deeper locations, where they were also more successful. Sediment composition played a key role in both habitat selection and hunting success, while seafloor disturbance only impacted the selection of foraging grounds. Given the expected changes in the hydrodynamic seabed morphology and sediment characteristics^22,23^ due to the strengthening of wind-induced currents and waves^22,23^ in southeastern Australia, shifts in the benthic habitat preferred by female AUFS are anticipated. These alterations could affect the availability and quality of their foraging habitats, potentially impacting their hunting success and leading to population-level consequences. To assess the population-level impact, further dedicated research across different sex and age classes is needed.

### Habitat selection

The selection of foraging habitat by predators involves identifying areas that increase the likelihood of successful prey capture. However, individuals face different challenges when selecting a proper foraging area, balancing the trade-offs between food availability, competition, and predation risk while dealing with environmental constraints^11,49^. In the present study, habitat selection by adult female AUFS was significantly influenced by bathymetry, sediment composition and disturbance of the seafloor. These results highlight the importance of environmental variables in shaping the foraging behaviour of female AUFS, emphasising the need to understand how changes in these parameters could influence their habitat selection.

Consistent with previous studies on other top predators (e.g. penguins, seals^19,45^), seafloor sediment composition influenced the habitat selection of AUFS. Sediment type and structure (e.g., reefs, gravel outcrops, flat seabed) are known to influence the distribution and abundance of benthic fish^50^ and play a key role in shaping the benthic habitat of key prey species for AUFS^21,29,33,51–53^. Female AUFS preferred benthic habitats with similar sediment composition and hydrodynamics to those selected by other predators, such as the yellow-eyed penguin, (*Megadyptes antipodes*)^19,54^. For example, yellow-eyed penguins selected sand ripple areas^54^, likely shaped by strong seafloor currents^55^, where their primary benthic prey, opal fish (*Hemerocoetes monopterygius*) and blue cod (*Parapercis colias*) are present. The selection of muddy-sandy areas and a higher seafloor disturbance index by female AUFS may be linked to the high-energy environment of the Bass Strait, which supports rich benthic communities^56^ and hosts a largely endemic invertebrate and benthic fish fauna^57^. Although little is known about the relationship between the distribution of the benthic fish and cephalopods that AUFS consume^32,33,58,59^ and the sandy-muddy areas, these sediment could likely reflect the habitat preferences of their prey or indicate favourable conditions that facilitate prey capture by AUFS. Understanding how AUFS’ prey species relate to hydrodynamics and sediment composition would help elucidate the influence of these factors on habitat selection by female AUFS.

Bathymetry has previously been reported as an influential factor in the habitat selection of female AUFS^38^, as well as for other demersal predators (e.g., gray seals, *Halichoerus grypus*^45^*,* yellow-eyed penguins^60^), consistent with the findings in the present study. Although individuals preferred greater depths in the present study, the most successful hunting occurred in moderately deep areas near the breeding colony in the central Bass Strait, where depths range from 60 to 80 m. This behaviour is likely driven by their central-place foraging strategy, which constraints individuals to forage within a limited area as they must return regularly to provision their offspring^61^. In addition, the foraging behaviour of the individuals from Kanowna Island may also be restricted by the high intraspecific competition, especially given the proximity of other AUFS breeding colonies^62,63^, which increases pressure from conspecifics. For example, females from the Seal Rock breeding colony have been recorded foraging in the northeast and central Bass Strait, potentially competing for resources with AUFS from Kanowna Island, which could lead to exclusion or avoidance behaviour by Kanowna seals^38^. Alternatively, this could reflect the distribution of the preferred prey of AUFS^64^, which has been reported to increase in diversity with depth in Bass Strait^56^.

Factors influencing foraging habitat selection have often been linked to acquiring high-quality resources ^12^, while the role of these areas in minimising predation risk remains poorly understood^10,61^. Female AUFS may be selecting these deeper foraging grounds to avoid predators, restricting their activity to safer areas with fewer predators as detected in other species^65^. For example, Cape fur seals (*A. p. pusillus*) reduce their activity in deep open water in favour of areas with abundant refugia (e.g. kelp forest^66^) to avoid white sharks (*Carcharodon carcharias*). In addition, pinnipeds are known to be major prey items for white sharks, which aggregate near fur seal breeding colonies in the shallow waters of South Australia^67,68^ ). Although the distribution of this predator and the predation risk faced by AUFS in the Bass Strait remain unknown, the presence of white sharks may drive the seals to select deeper areas farther from their breeding colonies. Gaining insights into the distribution of AUFS predators and the role of habitat features in mitigating predation risk would enhance our understanding of AUFS habitat selection.

### Hunting success

Seafloor sediment composition influenced hunting success in female AUFS. Individuals achieved higher hunting success rates in areas with greater rock composition and lower gravel concentrations. Both substrates could serve as orientation or navigational aids for seals, helping them to forage in areas with predictable prey, consistent with findings from other benthic-foraging top predators^19,69,70^. For instance, seafloor visual cues in yellow-eyed penguins reduce the randomness of their movement paths, guiding the individuals towards prey-rich areas and enhancing foraging efficiency^70^. Similarly, solid substrates may guide the benthic diving behaviour of AUFS, enabling the creation of memorised landscape maps that improve their hunting success. In addition, rocky substrates provide suitable habitats for AUFS’ key prey species, such as gurnards, leatherjackets^71^ and *Octopus* spp.^51^, facilitating foraging opportunities for the females that target these areas. While rocky regions enhance hunting success, gravel-rich areas can hinder AUFS’ ability to efficiently target and capture prey by reducing visibility and accessibility, making these habitats less ideal for seals for successful foraging successfully, despite their high species richness^72^. Understanding the importance of these habitats for the prey consumed by AUFS would be beneficial for comprehending the high foraging success associated with this type of seafloor sediment.

Hunting success was influenced by bathymetry, with success increasing in depths both at deeper regions (> 40 m) and at shallower depths (< 30 m). The increased success at greater depth may be attributed to the spatial and temporal predictability of their key prey items, supported by previous studies that have documented higher benthic species diversity with depth in Bass Strait^28,43,56^. Most foraging activity by individuals from Kanowna Island occurred within the central Bass Strait, where local bathymetry determined a maximum depth of around 60–80 m, suggesting bathymetry is more of a threshold predictor for foraging behaviour. A high probability of hunting success also occurred at shallower depths, such as near the coast of Tasmania and Flinders Island (40 S 148 03′ E), where rocky-reef habitats are prevalent^73^ and host a large abundance of demersal fish species^71,74^ that are known prey of AUFS^37,59,75^. In addition, these shallow waters near other breeding colonies (e.g. Wright Rocks, Double Island; Moriarty Rocks), offer suitable haul-out sites on land and may allow individuals from Kanowna Island to exploit profitable foraging grounds for longer periods^61,76^. However, despite the high success associated with these shallow benthic dives, they were observed in only 10 individuals and accounted for less than 1% of all dives. Such limited occurrence may reflect individual foraging specialisations in shallow rocky reef habitats, which is expected in benthic foragers^77,78^ and has been previously reported in AUFS^79^. Such specialised behaviour has been shown to enhance hunting success in generalist populations by reducing intraspecific competition, which may offer a buffer against environmental changes^80^, potentially benefiting female AUFS.

### Potential impacts of oceanographic changes

Numerous studies have established associations between climatic variations and both habitat use and hunting success in marine top predators (e.g. ^81,82^). Marine communities are undergoing negative impacts of anthropogenic climate change globally, including shifts in community composition and structure^83–86^. For example, fish assemblages in south-eastern Australia have shown significant changes in their diversity, abundance and distribution in response to rising sea temperatures^26,43^. Such changes may lead to the selection of different habitats and prey types, driven by the significant dietary plasticity that AUFS have shown in response to the rising sea temperature^32,75^. Their adaptability could be advantageous for female AUFS to maintain hunting success and resilience under the expected changes in the marine environment.

Projected climate change scenarios predict a strengthening of wind-induced currents and wave activity in the Bass Strait^22^. These changes are expected to disturb the seafloor and impact seabed mobility and erosion^22,23^, which could affect the smaller sediments preferred by female AUFS and influence their habitat selection. Such alterations in the benthic environment may affect species assemblages and result in shifts in the distribution of benthic prey consumed by AUFS, potentially influencing their habitat selection and hunting success. The magnitude and direction of these environmental changes, along with the specific benthic variables affected, will determine the extent of the AUFS’s response. Wind-induced currents can bring nutrient-rich waters closer to central Bass Strait^87^, potentially leading to shifts in prey preference that may impact hunting success in female AUFS. During periods of stronger winds^32^, certain prey species such as redbait occur more frequently in their diet when winds are stronger, which may influence hunting success and shift preferred foraging areas.

In summary, the present study revealed that habitat selection and hunting success in female AUFS are influenced by different environmental factors, with bathymetry and sediment composition playing critical roles. Individuals non-randomly selected foraging areas where they are not necessarily more successful. In addition, anticipated oceanographic changes that are expected to alter the sea floor characteristics and benthic communities could impact habitat selection and hunting success in adult female AUFS. To better understand how these potential changes may influence the AUFS population, further investigation is needed to explore how benthic environments may respond to the predicted climate-induced alterations and how these shifts may impact different age classes and sexes.

## Methods

### Ethics statement

All animal handling procedures for the present study were conducted in accordance with the regulations, guidelines and approval of the Deakin University Animal Ethics Committee (Approval A33/2004, A16/2008, A14/2011, B16/2014, B04/2017, B05/2020), and the Department of Sustainability and Environment (Victoria, Australia) Wildlife Research Permits (10,000,187, 10,000,706, 10,001,143, 10,001,672, 10,002,269, 10,005,362, 10,007,153, 10,008,286 and 10,005,848).

### Study site and animal handling

The study was conducted at Kanowna Island (39°10’S, 146°18’E; Fig. 1), northern Bass Strait (south-eastern Australia). The island hosts the third-largest breeding colony for the species^31^. During the autumn/winter months (May–August) of 2006–2021, adult female nursing pups were selected at random and captured with a modified hoop net (Fuhrman Diversified, Seabrook, Texas, USA). This sampling period corresponds to peak lactation for the species^88^ and, thus, the period of greatest nutritional demand for adult females provisioning young^89,90^. Individuals were then anesthetized for safe handling with isofluorane delivered via a portable gas vaporizer (Stinger, Advanced Anaesthesia Specialists, Gladesville, NSW, Australia) before being placed on a flat board for processing. For identification, uniquely numbered plastic tags (Super Tags, Dalton, Woolgoogla. Australia) were inserted in the trailing edge of each fore-flipper.

Individuals were then instrumented with a dive behaviour data logger (Mk06, Mk07, Mk08, Mk09, or Mk10, Wildlife Computers Ltd., Redmond, WA, USA) and a Fastloc GPS data logger (F1G Sirtrack Ltd, Havelock North, New Zealand), or a combined dive behaviour/Fastloc GPS data logger (MK10AF, Wildlife Computers Ltd). The devices were glued in series to the fur of the dorsal midline just posterior to the scapula using quick-setting epoxy resin (RS Components, Corby, UK). The dive behaviour and GPS data loggers were programmed to sample depth and location at 1 s or 5 s and 10 min, respectively. To assist with relocation for recapture, individuals were also instrumented with a VHF transmitter (Sirtrack Ltd., Havelock North, New Zealand) posterior to the other devices on the dorsal midline. The combined mass and cross-sectional area of the devices used were less than < 1% and, therefore, are likely to have had a negligible effect on the hunting success of individuals^91^. Following recovering from anaesthesia, individuals were released to resume normal behaviours. After one or more complete foraging trips, animals were recaptured using the previously described method and the data loggers were removed by cutting the fur beneath them.

### Data processing and analyses

The GPS data were filtered to remove erroneous locations and linearly interpolated along each foraging trip using the *trip* package version 1.8.7^92,93^ in the R statistical environment^94^. The dive behaviour records were first corrected for any potential drift in depth and the metrics of the dives (dive time, dive duration, maximum depth, bottom time, descent and ascent rate) were summarised using the *diveMove* package version 1.6.1^95^. The GPS tracks were then merged with the dive behaviour data to geolocate all dives. As fur seals may enter the water around colonies to thermoregulate^61^, only immersions > 8 h were considered foraging trips. In addition, to exclude dives in the proximity of the colony that may be conducted for predator avoidance^61^, all dives occurring within a 1 km buffer of the colony and haul-out sites were excluded.

Dives were classified as benthic or pelagic using the Intra-depth Zone (IDZ) method^96^ which considers sequential dives with maximum depths of ± 10% to be benthic. Subsequently, all pelagic dives, which represented a smaller proportion of the total dives, were excluded from further analyses. The geolocated benthic dives were then used as temporally independent locations for a habitat-selection analysis. The potential available habitat was determined as the at-sea area within a 300 km radius of the colony, restricted to the continental shelf (depth < 200 m), corresponding to the maximum observed distance from the colony attained by lactating female AUFS during foraging trips^38^. A 0.01° × 0.01° grid was then overlaid over this area and the number of dives within grid cells was compared to environmental variables.

An initial exploratory analysis was conducted to identify oceanographic variables influencing habitat selection (Table 2), applying an exclusion criterion to the ones with strong collinearity through the VIF method (*car* package version 3.1–2). The degree of habitat selection of AUFS was explored within the home ranges using *K*-select analysis adehabitatHS package version 0.3.17^38,97–99^. This analysis employs the marginality concept, evaluating the strength of habitat selection based on the mean difference between the environmental conditions of the home ranges (habitat uses) and habitat availability, which is the same for all animals design II^97^. The *K*-select provides a linear combination of environmental variables maximising mean marginality^97,100^. The orientation of the marginality vectors reveals the habitat selection of individuals, indicating whether they share habitat preferences^97^.

**Table 2:** Environmental variables of the habitat availability K-select model of Australian fur seals at 0.01(°) of resolution.

Environmental variables	Description	Source	Categories
Bathymetry	Digital sea floor elevation data (m)	GEBCO	10 m
Slope	Derived from the digital elevation data (°)	GEBCO	10°
Aspect	Derived from the digital elevation data (°)	GEBCO	10°
Mud	Interpolation of available online data (%)	^101^	10%
Sand	Interpolation of available online data (%)	^101^	10%
Gravel	Interpolation of available online data (%)	^101^	10%
Disturbance index	Sediment mobility number (% of time)	^101^	10%

Previous studies have demonstrated a correlation between dive profile metrics and prey capture, enabling the probability of whether an individual captured prey during a dive to be determined using dive behaviour data only^102^. In the present study, benthic dives were classified as successful (1, with at least one prey capture event) or unsuccessful (0, with no prey capture events) based on a previously derived relationship between dive metrics such as descent rate, ascent rate, bottom time, and the duration of previous dives and prey capture events measured using accelerometry (see Supplementary Material A1). The classified dives were then used in a habitat suitability model, to investigate the seafloor factors that impact hunting success, with successful dives as presence (1) and unsuccessful dives as absence (0).

To determine which environmental variables influenced hunting success, a habitat distribution model using a full subset generalised additive mixed model FSSgam^103^ was constructed with the *Fssgam* package version 1.11^103^. The same environmental covariates as used in the habitat selection analyses were included in the model, with the addition of depth and current velocity at the same resolution Bio ORACLE^104^. The function ‘generate.model.set’ was used to evaluate the importance of the variables and select the optimal model according to the Akaike’s Information Criterion AIC^105^. The FSSgam evaluated all viable candidate models, explored the relative importance of each potential indicator without overfitting, and identified the best-fitting model (s) influencing the successful dives^103^. To assess the viability of potential predictors, models with ΔAICc < 4 were considered supported^106^. All possible combinations of potential predictors were tested, with a limited maximum number of 3 explanatory variables and the number of knots for each parameter within a given model was restricted to 4. Environmental variables were transformed where necessary to meet the normality assumption required by the model. To investigate the relationship between the probability of dives being successful and the most representative covariates from the FSSgam, a generalized additive mixed effect model GAMM^107^ was fitted with individuals included as a random effect. Penalised thin plate regression splines were used to fit the smooth terms to all the predictor variables and the ‘gam. check’ function was used to determine the adequate number of knots in each smooth term^108^. A spatial prediction was generated to identify the optimal areas for successful dives using the GAMM predictors. The model was fitted by using *mgcv* package version 1.8–36^108^. All the statistical analyses were conducted in the R Statistical environment version 4.3.1^94^ and the maps were made with Quantum Geographic Information System (Qgis) version 3.30.1^109^.

## Supplementary Information

Supplementary Information.

## Data Availability

The datasets generated and/or analysed during the current study are available in the Zenodo repository.
