# Supplementary material for: Habitat selection and influence on hunting success in female Australian fur seals
Source: Sci Rep. 2024 Nov 6;14:26982. doi: 10.1038/s41598-024-78643-5 (PMC11541878; doi:10.1038/s41598-024-78643-5)
Supplement: Supplementary file 1 — Supplementary Information. [file 41598_2024_78643_MOESM1_ESM.pdf]

Supplementary material

A1: Equation to classify the 258,772 benthic dives of Australian fur seals into successful and unsuccessful.

Logit(P)= intercept + ndsr + ascr + dscr + btim + pbtim

Where P represents the probability of successful prey capture, *n.dsr* represents the descent rate of the next dive ($m.s^{-1}$), *ascr* represents the ascent rate of the current dive ($m.s^{-1}$), *dscr* represents the descent rate of the current dive duration time of the dive ($m.s^{-1}$), *btim* is the bottom time of the current dive (min) and *pbtim* represents the previous dive' bottom time (min; Mathews and Arnould unpublished data).

Table A2: Summary of adult female Australian fur seals sampled at Kanowna Island from 2006-2021**.**

Individual	Deployment date	Benthic dives (N)	Depth range (m)	Successful dives (%)
Seal 1	22/05/2021	5824	74.0-87.2	48.4
Seal 2	23/05/2021	5058	59.2-90.0	47.7
Seal 3	22/05/2021	6077	36.9-82.5	79.6
Seal 4	26/07/2010	1002	70.6-86.6	19.5
Seal 5	13/06/2006	519	75.3-91.9	27.7
Seal 6	8/07/2006	4326	72.0-87.7	62
Seal 7	9/06/2007	2652	39.3-87.3	32.6
Seal 8	19/07/2007	171	74.9-83.3	3.5
Seal 9	16/05/2008	406	57.7-82.0	28.1
Seal 10	21/05/2008	300	75.6-86.2	7
Seal 11	27/05/2008	346	69.3-78.6	6.4
Seal 12	28/05/2008	617	65.1-85.7	7.1
Seal 13	10/06/2008	1474	52.7-88.6	17.3
Seal 14	5/07/2008	2476	67.3-92.3	53
Seal 15	28/06/2008	1653	57.5-89.7	23.3
Seal 16	30/07/2008	571	75.5-89.0	87.9
Seal 17	31/07/2008	237	74.6-78.5	61.6
Seal 18	1/06/2009	1359	34.7-90.6	61.5
Seal 19	16/06/2009	1993	56.3-90.1	46.8
Seal 20	2/06/2009	4576	74.4-90.8	88.5
Seal 21	8/06/2009	508	74.5-85.6	4.9
Seal 22	8/06/2009	395	74.8-89.6	5.8
Seal 23	24/06/2009	701	75.6-87.9	18
Seal 24	11/06/2009	592	75.3-87.7	44.4
Seal 25	13/06/2009	1019	75.1-79.5	76.1
Seal 26	24/06/2009	1041	55.6-87.6	60.4
Seal 27	19/06/2009	2651	39.9-72.1	32.5
Seal 28	22/06/2009	386	51.8-84.9	45.9
Seal 29	30/06/2009	1138	17.8-53.1	9.1
Seal 30	2/07/2009	2068	43.0-87.6	6.7
Seal 31	2/08/2009	2230	55.6-90.0	59.6
Seal 32	27/07/2010	877	75.5-84.7	55.9
Seal 33	30/07/2010	237	75.7-78.4	32.1
Seal 34	1/08/2010	299	19.2-79.4	23.4
Seal 35	22/05/2011	272	37.3-84.9	15.4
Seal 36	15/05/2011	363	67.0-79.6	17.4
Seal 37	20/05/2011	1743	22.9-81.7	6.3
Seal 38	17/05/2011	591	74.8-88.2	65.5
Seal 39	28/05/2011	269	75.5-89.5	30.9

Seal 40	18/08/2018	745	73.2-90.1	5.5
Seal 41	28/05/2011	527	75.6-91.4	38.9
Seal 42	25/05/2019	8350	40.3-84.7	39.4
Seal 43	23/06/2011	3156	73.6-93.5	73.7
Seal 44	7/07/2011	3435	53.7-89.7	34.9
Seal 45	29/05/2012	2064	74.6-94.2	78.5
Seal 46	17/05/2012	1405	62.9-86.2	63.2
Seal 47	6/05/2018	552	74.7-88.5	31
Seal 48	18/05/2012	717	75.2-84.3	18.7
Seal 49	25/05/2012	608	64.5-89.2	28.3
Seal 50	26/06/2012	735	66.3-82.0	21.2
Seal 51	21/06/2012	133	51.8-74.3	7.5
Seal 52	1/07/2012	1030	46.3-74.5	22.2
Seal 53	11/07/2012	668	61.8-74.7	33.8
Seal 54	10/07/2012	413	75.5-87.1	85.5
Seal 55	19/07/2012	166	17.0-78.9	17.5
Seal 56	20/07/2012	1063	75.5-81.4	61.8
Seal 57	19/07/2012	306	79.1-94.9	67
Seal 58	25/07/2012	1165	25.1-80.6	15.2
Seal 59	9/08/2012	726	60.7-84.9	50
Seal 60	16/06/2013	6642	22.4-60.1	22.1
Seal 61	13/07/2013	1118	31.1-81.5	37.9
Seal 62	28/06/2013	598	36.3-94.3	73.4
Seal 63	31/05/2013	2132	48.7-92.4	81.7
Seal 64	6/07/2013	2302	70.3-91.9	93.2
Seal 65	25/06/2013	1705	0.2-82.3	54.1
Seal 66	5/07/2013	4166	71.4-91.7	58.4
Seal 67	11/06/2014	1903	55.0-82.7	32.7
Seal 68	27/06/2014	4948	75.5-86.4	58.8
Seal 69	19/06/2014	3352	59.7-87.4	42.2
Seal 70	26/06/2014	2846	74.8-93.2	71.6
Seal 71	24/07/2015	2032	39.7-78.2	26.8
Seal 72	18/07/2015	1372	49.4-76.9	38.6
Seal 73	10/07/2015	613	70.1-89.6	40.5
Seal 74	26/07/2016	295	72.2-81.2	24.4
Seal 75	28/08/2016	1741	66.6-82.6	13.1
Seal 76	29/07/2016	2074	75.0-82.0	16.4
Seal 77	29/05/2017	1152	67.0-87.2	35.2
Seal 78	10/05/2017	846	75.0-91.8	28.3
Seal 79	9/05/2017	671	75.6-82.0	52.3
Seal 80	21/05/2017	939	68.7-83.9	56.3
Seal 81	2/06/2017	1040	75.5-88.0	15.7
Seal 82	3/06/2017	216	75.3-89.3	51.9
Seal 83	4/06/2017	722	74.6-87.3	35.2
Seal 84	17/06/2017	6751	62.0-89.7	32.6

Seal 85	7/05/2018	63	75.9-92.0	7.9
Seal 86	16/08/2018	889	75.1-90.4	29.4
Seal 87	17/08/2018	5273	20.7-63.1	8.5
Seal 88	13/09/2018	3943	51.8-88.9	72.2
Seal 89	17/05/2019	11153	51.2-90.1	17.3
Seal 90	10/05/2019	8880	63.9-84.5	84
Seal 91	16/07/2019	9710	65.7-80.8	50.1
Seal 92	16/06/2019	4160	66.3-94.0	55.4
Seal 93	15/07/2019	8358	69.4-82.7	95.5
Seal 94	25/05/2019	4223	71.0-90.7	76.1
Seal 95	19/08/2020	7024	53.7-87.0	70.4
Seal 96	9/07/2020	3625	66.5-86.9	65.7
Seal 97	5/07/2020	3028	61.2-86.3	58.4
Seal 98	29/06/2020	3041	63.1-89.0	57
Seal 99	7/07/2020	3010	39.3-86.6	73
Seal 100	16/07/2020	2478	48.5-80.3	36.9
Seal 101	14/07/2020	1373	9.6-81.3	27.5
Seal 102	15/07/2020	3476	71.9-82.8	56.1
Seal 103	28/06/2020	3198	40.4-86.5	37.3
Seal 104	3/09/2020	4519	32.1-87.0	24.9
Seal 105	2/09/2020	7460	37.5-90.4	52.6
Seal 106	19/08/2020	2327	65.9-92.9	39.4
Seal 107	24/08/2020	3670	30.7-84.4	33.2
Seal 108	29/08/2020	559	71.0-89.0	24.3
Seal 109	28/08/2020	642	75.6-90.5	87.5
Seal 110	28/09/2020	2980	21.5-82.0	21.4
Seal 111	23/07/2021	4943	71.6-85.5	67.2
Seal 112	22/07/2021	2820	69.1-89.2	47.7
Seal 113	14/08/2021	3399	54.3-84.5	23.5
